# Root Perforations: Etiology, Diagnosis, Prognostic Factors, and Contemporary Repair Materials: A Narrative Review

**DOI:** 10.3390/dj14090574

**Published:** 2026-09-07

**Authors:** Desislava Tomlekova, Aleksandra Pecheva, Silviya Dimitrova

**Affiliations:** Department of Operative Dentistry and Endodontics, Faculty of Dental Medicine, Medical University of Plovdiv, 4002 Plovdiv, Bulgaria; desislava.tomlekova@mu-plovdiv.bg (D.T.); silviya.d.dimitrova@mu-plovdiv.bg (S.D.)

**Keywords:** root perforation, surgical treatment, conservative treatment

## Abstract

Root perforations represent a pathological communication between the root canal system and the external root surface and are among the most serious complications in endodontics. Their occurrence leads to bacterial contamination and inflammatory tissue destruction, resulting in resorption of dentin, cementum, and alveolar bone, thereby compromising the tooth’s long-term prognosis. **Objective:** The aim of this review is to summarize current data regarding the etiology, diagnosis, treatment strategies, and contemporary materials used in the management of root perforations, with particular emphasis on recent advances in non-surgical treatment. **Methods:** A narrative literature review was conducted using sources from PubMed, Scopus, Web of Science, Embase, CINAHL, and the Cochrane Library. After applying a predefined search strategy of inclusion and exclusion criteria, 60 scientific sources published between 2003 and 2026 were included. **Results:** Current evidence indicates that the treatment outcome depends primarily on the location and size, prompt perforation sealing, bacterial contamination, and selection of restorative material. Cone-beam computed tomography has improved diagnostic accuracy, while a non-surgical approach remains the preferred approach in most clinical situations. Mineral trioxide aggregate (MTA) continues to be the reference standard material due to its biocompatibility, bioactivity and sealing ability. New-generation calcium-silicate bioceramics offer improved physicochemical properties, while maintaining favorable biological properties. Regenerative materials have shown promising potential to enhance healing processes, but available evidence remains limited. **Conclusions:** Successful treatment of root perforations requires early diagnosis, timely repair of the defect, effective bacterial control, and use of bioactive materials with reliable sealing and regenerative properties. Modern calcium-silicate bioceramics have improved non-surgical treatment predictability, whereas regenerative approaches appear to be a promising alternative that requires further clinical studies to develop standardized therapeutic protocols.

## 1. Introduction

Root perforation is a communication between the root canal system and the supporting periodontal tissues or oral cavity [1,2,3]. These defects represent a significant complication in endodontic practice, with perforations identified as the second most common cause of endodontic treatment failure [1]. The creation of an abnormal pathway between the sterile root canal space and the contaminated external environment may have iatrogenic or pathological origin, but in general compromises the biological seal essential for successful endodontic therapy [4,5].

The clinical significance of root canal perforations extends beyond immediate treatment complications. Perforations can occur at various locations along the root surface—including the cervical, middle, and apical thirds, as well as the furcation area—each presenting unique diagnostic and therapeutic challenges [5,6]. The prognosis of perforated teeth depends on multiple interrelated factors, including the size and location of the defect, time elapsed between perforation and repair, degree of bacterial contamination, and the biological properties of the repair material used [4].

Historically, root canal perforations were associated with poor prognosis and often resulted in tooth extraction [1]. However, advances in diagnostic imaging, particularly cone beam computed tomography (CBCT), combined with the development of biocompatible repair materials such as mineral trioxide aggregate (MTA) and newer calcium silicate-based bioceramics, have significantly improved treatment outcomes [4,7,8]. Contemporary evidence suggests that with early diagnosis, appropriate material selection, and proper technique, many perforated teeth can be successfully managed and retained in function [9,10].

Current data indicate that the conservative approach is preferred due to the possibility of immediate sealing of the defect and preserving the tooth. The surgical approach is indicated in cases of failure of the conservative treatment, lack of orthograde access, persistent infection, or absence of a healing process [11].

Previously published reviews on root canal perforations have generally focused on a single dimension of the problem, such as etiology and classification, a single repair material, or surgical versus non-surgical decision-making, and several predate the introduction of contemporary calcium-silicate bioceramics and regenerative adjuncts. To date, no narrative review has jointly synthesized the prognostic factors influencing perforation outcome together with the comparative performance of traditional and current-generation repair materials. This narrative literature review examines the current state of knowledge regarding root canal perforations to provide clinicians with evidence-based guidance for prevention, diagnosis, and management of these challenging complications.

## 2. Materials and Methods

**Figure 1:**
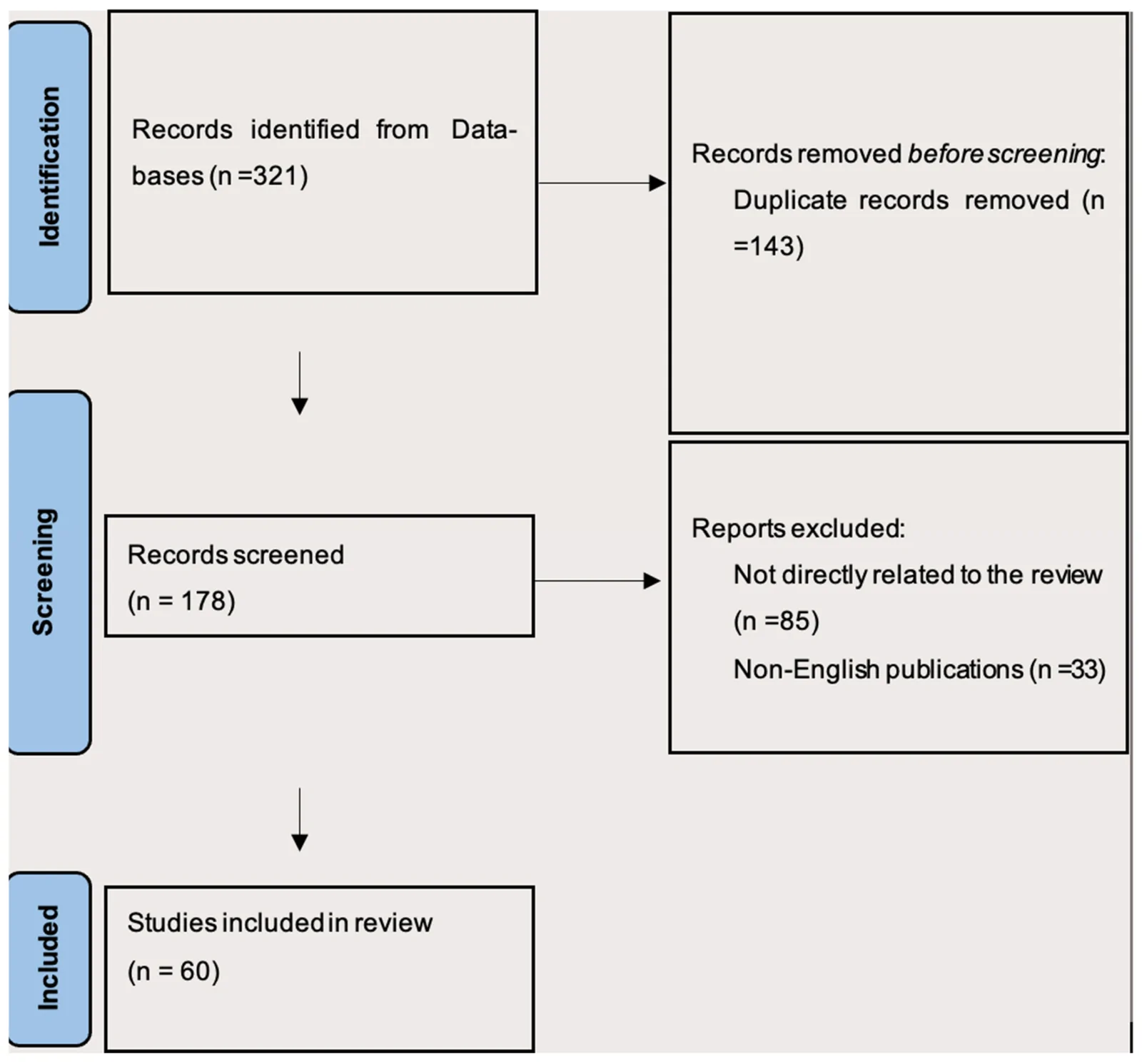
Simplified PRISMA-style diagram of the literature selection.

A narrative literature search was conducted through six electronic databases: Web of Science, Scopus, PubMed, CINAHL, the Cochrane Library, and Embase, covering publications from the past 23 years (2003–2026), with the exception of the classic classification paper by Fuss and Trope (1996), which was retained as a foundational reference despite predating this window. The last search was performed on 30 June 2026. The search strategy combined the terms (“root canal perforation” OR “iatrogenic perforation” OR “endodontic perforation”) AND (“etiology” OR “diagnosis” OR “classification” OR “prognosis” OR “repair” OR “treatment” OR “non-surgical treatment” OR “repair material”), adapted to the syntax of each database. The literature search identified 321 records. After removal of 143 duplicate records, 178 records underwent title and abstract screening by three independent reviewers (D.T., A.P., S.D.). Studies were included if they addressed the etiology, diagnosis, classification, prognostic factors, non-surgical or surgical management, or repair materials of root canal perforations in permanent teeth, and comprised randomized or non-randomized clinical trials, prospective or retrospective clinical studies, in vitro studies, animal studies, case reports or case series, and systematic or narrative reviews. Studies were excluded if they were non-English publications, editorials, conference abstracts, or did not directly address root canal perforations. Disagreements regarding study eligibility were resolved by discussion among the three reviewers until consensus was reached. After screening, a total of 60 records met the eligibility criteria and were included in the review. The selection process is illustrated in a simplified PRISMA-style flow diagram (Figure 1); as this is a narrative rather than a systematic review, the flow diagram is used solely as a transparent visual summary of the literature selection process and does not imply a systematic review methodology.

## 3. Results and Discussion

The evidence synthesized below spans multiple study designs, including in vitro, animal, case-report, and clinical studies, as well as reviews. Where a claim of comparative material performance is drawn from laboratory rather than clinical data, this is stated explicitly, as findings from these different levels of evidence are not directly interchangeable.

### 3.1. Etiology and Classification of Root Canal Perforations

#### 3.1.1. Iatrogenic Causes

Iatrogenic perforations represent the most common etiology of root canal perforations, occurring as procedural errors during different stages of endodontic treatment [4,12,13]. The literature identifies several critical procedural errors that predispose to perforation formation [6].

Access Cavity Preparation: Perforations during access cavity preparation typically result from failure to recognize tooth anatomy, improper angulation of the bur, or inadequate knowledge of pulp chamber morphology [14,15]. Calcified canals and teeth with significant coronal restorations present challenges, as clinicians may deviate from the natural canal path while searching for canal orifices [16]. Furcation perforations commonly occur when excessive dentin is removed from the pulp chamber floor during access preparation in multirooted teeth or in cases of obliteration [17,18].

Canal Instrumentation: Perforations during canal preparation often occur in curved canals when instruments are used with excessive force or when rigid instruments fail to follow the natural canal curvature [1,19]. The middle and apical thirds of curved roots are particularly vulnerable to strip perforations on the inner (concave) aspect of the curve, especially when large or inflexible instruments are used [11]. Ledge formation and subsequent attempts to bypass the ledge can also lead to lateral perforations [20,21].

Post Space Preparation: Post-related perforations occur during post space preparation when the operator deviates from the canal path, particularly in teeth with curved or dilacerated roots [22,23]. The use of oversized or improperly aligned post drills increases perforation risk [24]. Lateral perforations during post preparation are especially problematic in the cervical third of the root, where the defect communicates directly with the periodontal ligament space [25].

Retreatment Procedures: Endodontic retreatment carries elevated perforation risk due to altered canal anatomy, presence of calcifications, and difficulty in removing previous filling materials [26,27,28]. Attempts to bypass separated instruments or negotiate blocked canals can result in perforation [29]. The literature reports that retreatment cases require caution and often benefit from enhanced magnification and advanced imaging [28].

#### 3.1.2. Pathological Causes

Pathological perforations occur secondary to disease processes that compromise tooth structure. Internal resorption, when left untreated, can progress through the root canal wall to create a communication with the periodontal ligament [30]. External resorption, particularly cervical root resorption, may similarly perforate the root canal system [6]. Advanced carious lesions extending subgingivally can also create pathological perforations [25,30].

#### 3.1.3. Classification Systems

Root canal perforations are classified based on several parameters that influence treatment planning and prognosis [1,5].

- Location-based classification divides perforations into cervical third, middle third, apical third, and furcation perforations [5,6]. Cervical and furcation perforations generally carry a worse prognosis due to proximity to the oral environment and greater susceptibility to bacterial contamination [25].
- Time-based classification distinguishes between fresh and old perforations, according to the classification proposed by Fuss and Trope [31]. A fresh perforation is one recognized immediately at the time of occurrence, typically evidenced by sudden active bleeding from the canal, and is associated with minimal bacterial contamination. An old perforation is one diagnosed after a delay sufficient for bacterial colonization, inflammatory tissue reaction, and, in some cases, periodontal breakdown to have occurred; fresh perforations consistently offer a better prognosis due to reduced bacterial contamination and less extensive bone loss [4,31].
- Size-based classification categorizes perforations according to their diameter relative to a standardized endodontic instrument [31]. A small perforation is defined as one smaller than an ISO size #20 endodontic file, causing minimal mechanical damage to the surrounding tissue and offering a favorable prognosis when sealed promptly. A large perforation exceeds this size and is typically associated with significant destruction of the adjacent periodontal tissue, making an adequate hermetic seal more difficult to achieve; medium-sized defects fall between these two extremes. Larger defects present greater treatment challenges and reduced success rates [31].

### 3.2. Diagnosis of Root Canal Perforations

Early and accurate diagnosis of root canal perforations is critical for successful management and improved prognosis [4]. The literature emphasizes a multimodal diagnostic approach combining clinical signs, symptoms, and advanced imaging techniques.

Clinical Signs and Symptoms: Clinical indicators of perforation include sudden bleeding from the canal during instrumentation, pain during instrumentation in a previously asymptomatic tooth, and the presence of blood-tinged exudate [1,14,32]. Patients may report sensitivity or pain, particularly with furcation or cervical perforations that communicate with the periodontal ligament. However, many perforations, especially those occurring during access preparation or post space preparation, may be immediately apparent to the operator [4,7].

Radiographic Diagnosis: Conventional periapical radiography can reveal perforations when the defect is visible in the plane of the radiograph [1]. Radiographic signs include radiolucent areas adjacent to the root surface, disruption of the normal root contour, or extrusion of filling material beyond the root surface [19,27]. However, two-dimensional radiography has significant limitations, as perforations on the buccal or lingual aspects may not be visible, and overlapping anatomical structures can obscure small defects [33,34].

Cone Beam Computed Tomography (CBCT): CBCT has revolutionized the diagnosis of root canal perforations by providing three-dimensional visualization of tooth anatomy and perforation location [35]. The literature strongly supports CBCT use for perforation diagnosis, as it allows precise localization of the defect, assessment of the extent of bone loss, and evaluation of the relationship between the perforation and adjacent anatomical structures [33]. CBCT is particularly valuable for diagnosing perforations in complex cases, planning treatment approaches, and monitoring healing during follow-up [7].

Adjunctive Diagnostic Tools: Additional diagnostic aids include the use of magnification (surgical microscopes or loupes) to visualize perforation sites directly, electronic apex locators that may show altered readings when the file passes through a perforation, and the paper point test, where a paper point inserted into the canal becomes blood-stained at the level of the perforation [1,14].

### 3.3. Factors Affecting Prognosis

The prognosis of perforated teeth is multifactorial, with the literature consistently identifying several key determinants of treatment success [4].

Perforation Location: Location is one of the most critical prognostic factors [5,25]. Apical third perforations generally have the most favorable prognosis, as they are distant from the oral environment and surrounded by bone. Middle third perforations present an intermediate prognosis [5,36]. Cervical third and furcation perforations carry the poorest prognosis due to proximity to the gingival sulcus, direct communication with the periodontal ligament, and increased susceptibility to bacterial contamination from the oral cavity [25]. The literature reports that furcation perforations are particularly challenging, as they occur in an area with limited bone support and high bacterial exposure [10].

Time to Treatment: The interval between perforation occurrence and repair significantly impacts prognosis [4]. Immediate repair of fresh perforations prevents bacterial contamination of the perforation site and minimizes inflammatory damage to surrounding tissues [4]. Delayed treatment allows bacterial colonization, inflammatory cell infiltration, and progressive bone resorption, all of which compromise healing potential. Multiple studies emphasize that perforations should be managed as soon as they are diagnosed to optimize outcomes [4,14,30].

Perforation Size: Larger perforations present greater treatment challenges and reduced success rates [6,25,37,38]. Small perforations are easier to seal effectively and cause less disruption to the periodontal attachment apparatus [1]. Large defects may require more complex repair techniques, including the use of internal matrices to prevent extrusion of repair material into the periodontal space [29].

Bacterial Contamination: The presence and extent of bacterial contamination critically influence healing [4,39]. Perforations that remain exposed to the oral environment or saliva for extended periods develop bacterial biofilms that impede healing and promote chronic inflammation [1]. Prevention of bacterial infection through immediate sealing and maintenance of aseptic technique during repair are essential for success [4]. Estrela emphasizes that prolonged contact of the perforation defect with the oral microflora leads to persistent inflammation and compromises the healing of periodontal tissues [35]. The review by Alshehri et al. (2024) confirms the clinical significance of this mechanism, identifying bacterial contamination as a key prognostic factor and regarding it as a clinical risk factor that can influence the treatment protocol [11]. According to Gorni et al. (2022), the presence of clinical signs of communication with the periodontal environment increases the risk of long-term failure, highlighting the role of bacterial contamination in the prognosis of root perforations [40].

Repair Material Properties: The biological and physical properties of the repair material significantly affect outcomes [4,25]. Ideal repair materials should provide excellent sealing ability to prevent microleakage, demonstrate biocompatibility with periodontal tissues, resist displacement, and ideally promote regeneration of periodontal structures [4,7,8,41,42,43]. The advent of MTA and calcium silicate-based bioceramics has substantially improved perforation repair success rates compared to traditional materials [4,8,14].

Operator Experience and Technique: Practitioner experience and technical skill influence both the occurrence of perforations and the success of repair procedures [4]. Proper case selection, use of magnification, adherence to established protocols, and appropriate material handling all contribute to favorable outcomes [7,14].

### 3.4. Management Approaches

#### 3.4.1. Non-Surgical Management

Non-surgical management represents the first-line approach for most root canal perforations and is indicated when the perforation is accessible through the root canal system, the periodontal attachment is intact, and there are no significant complications such as extensive bone loss or periodontal communication [1,4,44,45].

Indications for Non-Surgical Approach: In non-surgical management, the perforations can be accessed and sealed through the root canal, particularly when diagnosed early [32]. Non-surgical repair is preferred for apical third perforations, small to moderate-sized defects, when the perforation site can be adequately isolated and visualized, and when the perforation is located in an area that would be anatomically difficult to access surgically [1,5]. Fresh perforations without significant bacterial contamination are most suitable for non-surgical repair [4,37].

Non-Surgical Repair Protocol: The non-surgical repair protocol involves several critical steps [4,7]. First, the perforation site must be located precisely, often with the aid of magnification and CBCT imaging [7,33,45]. The area is then thoroughly debrided and disinfected using appropriate irrigants, typically sodium hypochlorite, to eliminate bacteria and debris [14,20]. An internal matrix may be placed to prevent extrusion of the repair material into the periodontal space, particularly for larger perforations [29]. The repair material is then carefully placed into the perforation site using appropriate carriers and condensed with suitable instruments [4,7]. Multiple studies describe the use of MTA carriers or specialized pluggers for precise material placement [4,14].

Technical Considerations: Successful non-surgical repair requires adequate moisture control while maintaining the hydration necessary for MTA and bioceramic materials to set properly [8]. The literature emphasizes the importance of placing the repair material in small increments to ensure complete filling of the defect without voids [4,7]. Operating microscopes or loupes significantly enhance visualization and precision during repair procedures [14]. Following perforation repair, the remainder of the root canal system should be cleaned, shaped, and obturated according to standard endodontic protocols [4,28].

Case Selection: Non-surgical management is most successful when applied to appropriate cases [1,5]. Contraindications include perforations with extensive bone loss, significant periodontal involvement, inability to achieve adequate isolation, or perforations that cannot be accessed through the canal system [18,24].

#### 3.4.2. Surgical Management

Surgical management is indicated when non-surgical approaches are not applicable or have previously failed [18,24]. Surgical intervention may be necessary for perforations that cannot be accessed through the root canal, cases with extensive periodontal pathology, perforations with significant bone loss, or when non-surgical repair has been unsuccessful [1,18].

Surgical Approaches: Surgical management typically involves flap elevation to expose the perforation site, direct visualization and debridement of the defect, placement of biocompatible repair material from the outer root wall, and possible guided tissue regeneration procedures [18,24]. The literature describes various surgical techniques, including traditional periradicular surgery, intentional replantation with extraoral repair, and regenerative procedures using bone grafts and membranes [9,24].

Intentional Replantation: Intentional replantation represents a conservative surgical alternative for cases where conventional surgical access is difficult. After a tooth extraction, the extraoral perforated wall is repaired, and immediate replantation is done. The literature reports successful outcomes with intentional replantation when performed carefully with minimal trauma to the periodontal ligament [9].

Regenerative Procedures: Advanced surgical approaches may incorporate regenerative techniques to restore lost periodontal support [24]. Guided tissue regeneration using barrier membranes, bone grafting materials, or growth factors can enhance healing and bone regeneration around perforation sites [9,24]. These techniques are particularly relevant for furcation and cervical perforations with significant bone loss [10,46].

### 3.5. Repair Materials

Manufacturer information for the commercial materials discussed in this section is as follows: MTA Angelus and MTA Repair HP (Angelus, Londrina, PR, Brazil); Bio-C Repair (Angelus, Londrina, PR, Brazil); ProRoot MTA (Dentsply Sirona, Charlotte, NC, USA); Biodentine (Septodont, Saint-Maur-des-Foss\u00e9s, France); NeoPUTTY (Avalon Biomed, Bradenton, FL, USA); BioAggregate (DiaDent Group International, Burnaby, BC, Canada); EndoSequence Root Repair Material\u2013 ERRM (Brasseler USA, Savannah, GA, USA); and Endoseal MTA (Maruchi, Wonju, Republic of Korea). 

#### 3.5.1. Traditional Materials

Historically, various materials have been used for perforation repair, each with specific advantages and limitations [1]. Traditional materials include amalgam, zinc oxide eugenol, calcium hydroxide, glass ionomer cement, composite resin, and gutta-percha (Table 1) [4,25,30].

Today, the use of these traditional materials is either purely historical or restricted to specific clinical situations in which no contemporary bioceramic repair material is available.

#### 3.5.2. Contemporary Materials

##### Mineral Trioxide Aggregate (MTA)

Mineral trioxide aggregate is the gold standard for perforation repair since its introduction, demonstrating superior properties compared to traditional materials [4,7,8,14,30]. It consists of fine hydrophilic particles of tricalcium silicate, tricalcium aluminate, tricalcium oxide, and small amounts of mineral oxides, with bismuth oxide providing radiopacity [4]. Upon mixing with water, MTA forms a colloidal gel that sets into a hard structure with a high pH of 12.5 [4]. The material demonstrates low solubility, adequate compressive strength, and excellent dimensional stability [4,8]. MTA exhibits superior sealing properties compared to traditional repair materials [4]. Studies demonstrate that MTA leaks significantly less than amalgam and super EBA when placed in root-end preparations [4]. The material’s expansion during setting and its hydrophilic nature contribute to its excellent marginal adaptation and sealing ability [8,14].

One of the most beneficial properties of MTA in the case of root perforations is its biocompatibility and bioactivity. It demonstrates exceptional biocompatibility with periodontal and periapical tissues [4,7,8]. The material is less cytotoxic and non-mutagenic compared to traditional materials [4]. Importantly, MTA actively promotes hard tissue formation, including cementogenesis and osteogenesis [4,7,8]. The literature reports that MTA allows cementum overgrowth on its surface, facilitating periodontal regeneration [4,14]. The high pH of MTA provides antimicrobial properties and creates an environment conducive to healing [8]. Therefore, the material is indicated for repair of lateral root perforations, furcation perforations, strip perforations, and apical perforations [4,7,14,30,44,46,47].

Despite its excellent properties, MTA has some limitations, including long setting time (2–4 h), difficult handling characteristics, high cost, potential for tooth discoloration (particularly with gray MTA), and technique sensitivity [8,14]. The material’s granular consistency can make precise placement challenging, especially in small perforations [7].

The literature extensively documents successful outcomes with MTA for perforation repair [4,7,9,12,14,44,46,47]. Case reports and clinical studies demonstrate asymptomatic healing, bone regeneration, and long-term tooth retention following MTA repair of perforations at various locations [4,7]. Follow-up periods ranging from months to years show favorable clinical and radiographic outcomes, with resolution of periradicular pathology and maintenance of periodontal health [9,19,48,49].

##### Calcium Silicate-Based Bioceramics

Newer calcium silicate-based bioceramics have been developed to address some of MTA’s limitations while maintaining or improving its favorable biological properties [8,12,14,50]. Several studies compared the marginal adaptation and porosity of three calcium-silicate materials (ProRoot MTA, NeoPutty and Biodentine) used to repair furcation perforations using micro-CT analysis. The results showed that ProRoot MTA and NeoPutty demonstrated better marginal adaptation and lower porosity compared to Biodentine, suggesting a better ability to seal the perforation defect. The authors concluded that the physical properties of calcium-silicate materials, especially low porosity and good adaptation to dentinal walls, are essential for limiting microleakage and for a favorable prognosis in the treatment of perforations. Recently introduced bioceramic materials such as Bio-C show promising healing of lateral perforations, with favorable healing of the periapical tissue and predictable sealing, although longer-term follow-up is still needed to confirm these results [14].

Yalniz H. et al. compared four bioceramic materials for the repair of furcation perforations—MTA Angelus^®^, EndoSequence Root Repair Material (ERRM), Biodentine™, and BioAggregate—evaluating them via micro-CT analysis of porosity. ERRM exhibited the lowest total porosity among the tested bioceramic materials, suggesting superior sealing properties and potentially higher resistance to microleakage [50]. In a related in vitro study, better marginal adaptation was similarly associated with reduced microleakage, supporting a more effective hermetic seal [51].

In an in vitro study by Özel and Erişen, ERRM demonstrates the best adhesion stability in an acidic environment; however, prospective clinical studies are needed to investigate the long-term healing of periradicular tissues [52].

Baralay et al. (2022) found that, regarding the ability to limit bacterial microleakage during the repair of furcation perforations, MTA Angelus, Biodentine, and Endoseal have a similar effect, with no statistically significant differences [53].

Biodentine: Biodentine is a calcium silicate-based material with improved handling characteristics compared to MTA [47]. The material features a shorter setting time (approximately 12 min), better mechanical properties, and a more user-friendly consistency [47]. Biodentine demonstrates biocompatibility, bioactivity, and sealing ability comparable to MTA [8]. Clinical studies report successful outcomes with Biodentine for furcation perforation repair, with favorable healing at 2-year follow-up [47].

Bio-C Repair: Bio-C Repair represents a newer generation of ready-to-use bioceramic repair material. The material is supplied in a premixed, injectable form that eliminates the need for manual mixing and improves handling [14]. It demonstrates biocompatibility, radiopacity, and dimensional stability. Case reports document successful perforation repair with Bio-C Repair, showing clinical and radiographic healing [14].

Other Calcium Silicate Materials: The literature describes various other calcium silicate-based materials for perforation repair, including calcium-enriched materials and different proprietary formulations [12,22,30]. These materials generally share the favorable biological properties of calcium silicate chemistry, including biocompatibility, bioactivity, and capacity to promote hard tissue formation [8,12,22].

Regeneration materials in combination with Calcium Silicate Materials: In recent years, the potential synergistic effect of the combined use of bioregenerative and bioceramic materials has also been investigated. Kim et al. (2021) showed that combining calcium-silicate cements with EMD enhances the viability of human bone marrow-derived mesenchymal stem cells, increases alkaline phosphatase activity, and stimulates mineralization compared to the use of calcium-silicate materials alone. The results suggest that EMD can potentiate the osteogenic and regenerative effects of bioceramic cements [54]. After analyzing experimental and clinical data, Wang et al. (2018) conclude that EMD possesses promising properties as a bioactive material for the treatment of endodontic defects—including root perforations—particularly when periodontal tissue regeneration is required [55].

In addition to Enamel Matrix Derivative, attention is focused on the use of autologous platelet concentrates—including PRF and Concentrated Growth Factors (CGF)—which, thanks to their bioactive molecules, can support periodontal tissue regeneration during the repair of root perforations. Teja et al. (2021) report favorable healing following the combined use of MTA and Platelet-Rich-Fibrin (PRF) for a non-surgical treatment of a strip perforation, with PRF promoting periodontal tissue regeneration and MTA ensuring the sealing of the defect [56]. Mohamed et al. demonstrate in an experimental animal model that the use of PRF or concentrated growth factors as an internal matrix, in combination with MTA, promotes periodontal tissue regeneration and the formation of mineralized tissue [57].

Despite these encouraging findings, the evidence base for combining regenerative materials with contemporary calcium-silicate cements remains limited to in vitro, animal, and isolated case-report-level studies, with no controlled clinical trials currently available to support their routine combined use [54,55,56,57].

Ratings in this table represent a qualitative synthesis of the mixed in vitro, animal, and clinical evidence discussed in Section 3.5.2, rather than a formal evidence grading; “gold standard” reflects historical and current widespread clinical use of MTA rather than superiority on every parameter tested [4,7,8,14,30,52].

#### 3.5.3. Comparative Analysis of Repair Materials

The literature provides substantial evidence comparing different repair materials for perforation management (Table 2) [1,4,8,22,25].

As in Table 3 ratings summarize the cited literature qualitatively rather than a formal evidence grading, and largely reflect laboratory- and case-level rather than comparative clinical data.

### 3.6. Clinical Outcomes and Success Rates

Successful perforation repair is characterized by resolution of clinical symptoms, absence of pain or swelling, maintenance of periodontal probing depths within normal limits, and radiographic evidence of bone regeneration [4,19]. Radiographic healing typically shows progressive reduction in periradicular radiolucency and restoration of normal trabecular bone pattern [19,48]. Complete radiographic healing may require 6 to 24 months, depending on the size of the initial defect [9,19].

The literature provides substantial evidence regarding clinical outcomes and success rates for perforation repair, with outcomes varying based on perforation characteristics, treatment approach, and repair material used [1,4,11,37].

Material-dependent outcomes: Contemporary studies using MTA and calcium silicate-based bioceramics report high success rates for perforation repair [4]. Case series and clinical studies document success rates ranging from 80% to 95% for appropriately selected cases treated with modern bioceramic materials [4,11]. These figures should be interpreted with caution, as the underlying studies are heterogeneous in design, perforation location, follow-up duration, and definition of success, and few are directly comparable. These success rates represent significant improvement compared to historical outcomes with traditional materials [1].

It continues to be considered the reference material (the “gold standard”), but in recent in vitro comparative studies, it is often outperformed by Bio-C Repair or Biodentine in terms of laboratory-tested sealing ability or mechanical properties; clinical comparative data remain limited [52].

A comparative in vitro study found that both Biodentine and MTA Repair HP significantly increase the fracture resistance of roots with simulated perforations; although Biodentine also demonstrated high efficacy, MTA Repair HP provided greater mechanical stability under the experimental conditions [59].

In an in vitro glucose-penetration study, Biodentine provided the best marginal seal for the repair of furcation perforations, followed by Bio-C Repair, while MTA demonstrated the poorest sealing ability among the three materials tested, suggesting Biodentine as the preferred choice when minimizing microleakage is the primary criterion [58].

Location-Specific Outcomes: Success rates vary significantly based on perforation location [1,11]. Apical third perforations demonstrate the highest success rates, often exceeding 90% with proper management [33]. Middle third perforations show intermediate success rates [1]. Furcation and cervical third perforations present greater challenges, with success rates typically ranging from 60% to 80%, depending on the extent of bone loss and bacterial contamination [10,30].

Sarao S.K. et al. confirm that the location of the perforation is one of the key prognostic factors. Perforations situated in the furcation and crestal bone areas carry a less favorable prognosis due to the higher risk of bacterial contamination and periodontal damage, whereas apical and coronal perforations generally have a better treatment outcome if sealed promptly [2].

Alshehri M.M. et al. (2024) conclude that treatment success depends significantly on the location. Coronary perforations have a favorable prognosis in the absence of periodontal damage. Crestal/cervical perforations carry the poorest prognosis due to communication with the gingival sulcus. Apical perforations have a favorable prognosis given adequate preparation and sealing, whereas for furcation perforations, the prognosis depends on early sealing and the extent of bone damage [11].

Time-Dependent Outcomes: Immediate repair of fresh perforations yields significantly better outcomes than delayed treatment of old perforations [4,37]. Studies demonstrate that perforations repaired within the same appointment as their occurrence show superior healing compared to those repaired days or weeks later [4]. The literature emphasizes that each day of delay allows progressive bacterial contamination and bone resorption, compromising healing potential [37,39].

Long-Term Follow-Up: Case reports and longitudinal studies document sustained success with perforation repair over extended follow-up periods [9,46,47]. Reports describe successful outcomes at 1-year, 2-year, 5-year, and even 10-year follow-up, with maintained periodontal health, absence of symptoms, and radiographic evidence of bone healing [9,46,48]. These long-term data support the durability of modern perforation repair techniques when properly executed [19].

## 4. Conclusions

Root perforations remain a significant but generally manageable challenge in endodontic practice: outcomes depend primarily on early diagnosis, prompt sealing, perforation location and size, and selection of a biocompatible, well-sealing repair material. Mineral trioxide aggregate and newer calcium silicate-based bioceramics have improved sealing ability, biocompatibility, and regenerative potential compared with traditional materials and are increasingly regarded as the standard of care, with non-surgical treatment as the first-line approach and apical perforations carrying the most favorable prognosis. Of the 60 publications included in this review, approximately 46 discussed prognostic factors, 34 addressed repair materials, and 18 compared contemporary calcium-silicate biomaterials; the literature is unanimous that non-surgical management and perforation location are principal determinants of outcome, whereas evidence on a single “best” repair material and on regenerative adjuncts combined with calcium-silicate cements remains limited and continues to evolve. With a systematic approach grounded in prevention, early diagnosis, and meticulous technique, root canal perforations do not necessarily lead to tooth loss, and favorable long-term outcomes preserving tooth function can be achieved.

## Figures and Tables

**Table 1:** Traditional materials for root perforation repair.

Material	Advantages	Limitations	Current Clinical Role
Amalgam	Easy handling; good radiopacity [4]	Poor sealing ability; lack of biocompatibility; corrosion; tooth and soft tissue discoloration; no bioactivity or regenerative potential	Largely obsolete; replaced by bioactive calcium silicate materials [1,25]
Calcium hydroxide	Antimicrobial activity; high pH; stimulates hard tissue formation [25]	High solubility; poor long-term seal; resorption over time; low mechanical strength	Mainly used as an intracanal medicament or temporary dressing rather than as a definitive perforation repair material
GIC	Biocompatible; chemical adhesion to dentin; fluoride release; suitable for cervical and subgingival defects [4,30]	Inferior sealing ability; moisture sensitivity during setting	May be used for selected cervical perforations; no longer regarded as the material of choice
RMGIC	Improved mechanical properties and sealing ability compared with conventional GIC; good moisture tolerance [4,30]	Still inferior to calcium silicate-based materials in biological performance and regenerative potential	Limited use, restricted to a small number of selected clinical situations

**Table 2:** Comparison between currently available calcium silicate-based repair materials.

Properties	MTA	Biodentine	Bio-C Repair
Bioactivity	excellent [4,7,8]	excellent [8,47]	excellent [14]
Biocompatibility	excellent [4,7,8]	excellent [8]	excellent [14]
Sealing ability	excellent [4,8]	excellent (in vitro) [8,58]	excellent (in vitro) [14,51]
Setting time	Long	Fast	fast
Handling	Hard	Moderate	easy
Risk of discoloration	High	No	no
Moisture required for setting	Yes	No	No
Premixed formulation	No	No	yes
Clinical evidence	Extensive (clinical + in vitro) [4,7,9,12,14,44,46,47]	Moderate-high (mostly in vitro; some clinical) [47]	Limited but growing (case reports; limited in vitro) [14]
Current recommendation	Gold standard	Excellent alternative	Promising new-generation material

**Table 3:** Evolution of materials for root perforation repair.

Material	Bioactivity	Sealing Ability	Biocompatibility	Clinical Limitations	Current Status
Amalgam	No	Low	Low	Discoloration; leakage; corrosion	obsolete
Zinc-oxide eugenol cement	No	Moderate	Moderate	Solubility; weak mechanical properties	Rarely used
Calcium hydroxide	Moderate	Low	Good	High solubility; poor long-term seal	Temporary medicament
GIC	No	Moderate	Good	Lower sealing ability than bioceramics	Limited indications
RMGIC	No	Moderate-good	Good	Limited regenerative capacity	Selected indications
Composite resin	No	Good (when isolation is adequate)	Moderate	Technique-sensitive; polymerization shrinkage	Limited use
Gutta-percha	No	Poor	good	No adhesion; no regeneration	Historical use
MTA	Excellent [4,8]. These materials stimulate cementogenesis and osteogenesis, facilitating periodontal regeneration [4,14]. Traditional materials lack this bioactive property and function primarily as inert barriers [1,25]	Excellent [4,8,22]	Excellent [4]. Traditional materials such as amalgam and zinc oxide eugenol demonstrate greater cytotoxicity and less favorable tissue reactions [1]	Long setting time, discoloration; difficult handling	Gold standard
Biodentine	Excellent (in vitro; limited clinical) [8,47]	Excellent (in vitro) [8,58]	Excellent (in vitro) [8]	Higher cost	Preferred contemporary material
Premixed bioceramics (ERRM, Bio-C-Repair, etc.)	Excellent (in vitro) [14,50]	Excellent (in vitro) [50,51]	Excellent (in vitro; limited clinical) [14]	Limited long-term evidence for some products	Current generation

## Data Availability

The original contributions presented in this study are included in the article. Further inquiries can be directed to the corresponding author.

## Abbreviations

The following abbreviations are used in this manuscript:

MTA	Mineral Trioxide Aggregate
CBCT	Cone Beam Computed Tomography
GIC	Glass Ionomer Cement
RMGIC	Resin-Modified Glass Ionomer Cement
EMD	Enamel-Matrix-Derivate
PRF	Platelet-Rich-Fibrin
CGF	Concentrated Growth Factors
